# Factor Analysis Influencing Postoperative Hospital Stay and Medical Costs for Patients with Definite, Suspected, or Unmatched Diagnosis of Acute Cholecystitis according to the Tokyo Guidelines 2013

**DOI:** 10.1155/2016/7675953

**Published:** 2016-04-28

**Authors:** Aoi Hayasaki, Koji Takahashi, Takehiro Fujii, Koji Kumamoto, Koji Fujii, Eiichi Matsumoto, Shigeki Miyahara, Tsukasa Kusuta, Yoshinori Azumi, Shuji Isaji

**Affiliations:** ^1^Department of Surgery, Ise Red Cross Hospital, 1-471-2 Funae, Ise, Mie Prefecture 58512, Japan; ^2^Department of Hepatobiliary Pancreatic and Transplant Surgery, Mie University Graduate School of Medicine, 2-174 Edobashi, Tsu, Mie Prefecture 514-8507, Japan

## Abstract

*Purpose*. To identify significant independent preoperative factors influencing postoperative hospital stay (PHS) and medical costs (MC) in 171 patients who underwent cholecystectomy for benign gallbladder diseases and had definite, suspected, or unmatched acute cholecystitis (AC) diagnosis according to the Tokyo Guidelines 2013 (TG13).* Methods.* The 171 patients were classified according to the combination of diagnostic criteria including local signs of inflammation (A), systemic signs of inflammation (B), and imaging findings (C): A+ B+ C (definite diagnosis, *n* = 84), A+ B (suspected diagnosis, *n* = 25), (A or B) + C (*n* = 10), A (*n* = 41), and B (*n* = 11).* Results*. The A+ B + C and (A or B) + C groups had equivalent PHS and MC, suggesting that imaging findings were essential for AC diagnosis. PHS and MC were significantly increased in the order of severity grades based on TG13. Performance status (PS), white blood cell count, and severity grade were identified as preoperative factors influencing PHS by multivariate analysis, and significant independent preoperative factors influencing MC were age, PS, preoperative biliary drainage, hospital stay before surgery, albumin, and severity grade.* Conclusion*. PS and severity grade significantly influenced prolonged PHS and increased MC.

## 1. Introduction

Laparoscopic cholecystectomy (LC) has become the standard treatment for acute cholecystitis (AC) [1]; however, when AC becomes more severe, it increases the risk of major complications such as bile spillage, major bleeding, common bile duct injury, and bowel injury [2], resulting in prolonged postoperative hospital stay (PHS) [3]. The clinical features of patients with AC differ by disease severity; therefore, AC severity assessment is important in providing suitable medical management for each patient. Under these circumstances, the Evidence-Based Practice Guidelines for the Management of Acute Cholangitis and Cholecystitis were published in Japanese for the first time in 2005 (JG05) [4]. The Tokyo Guidelines 2007 were the first international practical guidelines (TG07) [5]. After the Revision Committee for TG07 performed multi-institutional studies and collected cases of acute cholangitis, AC, and noninflammatory biliary disease [6], TG07 was revised as the Tokyo Guidelines 2013 (TG13) [7].

LC is recognized as a cost-effective treatment for patients with AC compared with open cholecystectomy [8–10], and various factors influencing length of hospital stay including PHS and medical costs (MC) during hospitalization have been studied [3, 11, 12]. Identification of significant independent factors that affect PHS and MC in patients with AC not only is beneficial for the quality management of patient medical treatment but also is useful to assess severity grading. No previous studies have focused on MC as an index to evaluate AC severity grading, although many studies of AC severity indices have used perioperative complications, major organ damage, and hospital stay [3, 13–16]. For the purpose of reducing MC in Japan, a new payment system based on the diagnosis procedure combination/Per-Diem Payment System (DPC/PDPS) [17] was developed and introduced in a medical treatment fee system in 2003 [18], and the DPC database recently became a feasible tool for the evaluation of care processes that can provide useful information contributing to improved medical treatment quality [19].

The AC diagnostic criteria consist of three major factors: local signs of inflammation (A), systemic signs of inflammation (B), and imaging findings (C). It is noteworthy that C is essential for the definite diagnosis of AC and that AC is suspected when A and B are present [20]. According to these criteria, however, some patients with C who are elderly and/or have dementia or paralysis are not definitively diagnosed with AC because they do not present with A or B [21]. Fever and elevated white blood cell count are not usually observed in elderly patients with AC because of their decreased antistress capacity [22, 23]. Recently, Zhang et al. [24] reported the significance of ultrasound examination in the elderly with AC because ultrasound score could accurately determine AC severity in the elderly. Therefore, the TG13 diagnostic criteria should be reassessed, especially in the elderly.

The mortality rate in patients with AC in the 1960s was comparatively high at 4% [25, 26]. However, since the 2000s, this rate decreased to <1% with improvements in medical treatment [27–29]. Therefore, mortality is no longer suitable as an indicator of AC prognosis in clinical practice, and we focused on PHS and MC as the clinical outcomes that should reflect AC severity. The aim of the present study was to identify the significant independent preoperative factors influencing PHS and MC in patients with definite, suspected, or unmatched AC diagnoses according to TG13, paying attention to the elderly and those with dementia who showed AC findings on imaging but lacked local or systemic signs of inflammation.

## 2. Materials and Methods

We reviewed the clinical database of 259 consecutive patients who underwent simple cholecystectomy for benign gallbladder diseases such as AC, adenomyomatosis, and benign polyps from January 2012 to July 2013 at Ise Red Cross Hospital. We excluded 57 patients who were treated conservatively at the first admission and operated after readmission and 31 without symptoms and/or findings of AC. Thus, we included 171 patients in the present study.

In our hospital, every in-hospital patient has his or her physical performance status assessed on admission by a nurse using the Criteria for Evaluating the Degree of Independence of Disabled Elderly Persons in Performing Activities of Daily Living [30], and it is recorded with the ranks of J, A, B, and C in electronic medical records. Therefore, we used these records for the assessment of physical performance, and these ranks could be translated into the performance status (PS) defined by the Eastern Cooperative Oncology Group [31], because ranks J, A, B, and C are compatible with PS 0 and 1, PS 2, PS 3, and PS 4, respectively. We defined PS 0, 1, 2, and 3 as “better” and PS 4 as “poor” in the present study.

### 2.1. AC Diagnostic Criteria and Severity Grading

We amassed diagnostic findings from electronic medical records and classified the patients with A (local signs of inflammation) + B (systemic signs of inflammation) + C (imaging findings) as definite diagnosis and those with A + B as suspected diagnosis based on the TG13 diagnostic criteria for AC (see the following list).

*TG13 Diagnostic Criteria for AC*. The criteria are as follows (TG13: Tokyo Guidelines 2013, AC: acute cholecystitis, RUQ: right upper quadrant, CRP: C-reactive protein, and WBC: white blood cell):

- local signs of inflammation and so forth:
  (1) Murphy's sign,
  (2) RUQ mass/pain/tenderness;
- systemic signs of inflammation and so forth:
  (1) fever,
  (2) elevated CRP,
  (3) elevated WBC count;
- imaging findings:
   imaging findings characteristic of AC.
- Suspected diagnosis: one item in A + one item in B.
- Definite diagnosis: one item in A + one item in B + C.

In the present study, new combinations—that is, the unmatched diagnoses of (one item in A or one item in B) + C and one item in A, B, or C—were added. The following list shows AC severity grades defined by TG13, which, in principle, should be employed for patients with definite AC diagnosis. In the present study, however, this severity grade was employed in the 171 patients with definite diagnoses as well as those with suspected or unmatched diagnosis as a matter of convenience.

*AC Severity Assessment by TG13*

*Grade III.* Grade III is associated with dysfunction of any one of the following organs/systems:

(1) Cardiovascular dysfunction^*∗*^.
(2) Neurological dysfunction^*∗*^.
(3) Respiratory dysfunction^*∗*^.
(4) Renal dysfunction^*∗*^.
(5) Hepatic dysfunction^*∗*^.
(6) Hematological dysfunction^*∗*^.

*Grade II.* Grade II is associated with any one of the following conditions:

1. Elevated WBC (>18,000/mm^3^).
2. Palpable tender mass in the right upper abdominal quadrant.
3. Duration of complaints > 72 h.
4. Marked local inflammation (gangrenous cholecystitis, pericholecystic abscess, hepatic abscess, biliary peritonitis, and emphysematous cholecystitis).

*Grade I.* Grade I does not meet the criteria of “Grade III” or “Grade II” AC:

 AC: acute cholecystitis, TG13: Tokyo Guidelines 2013, and WBC: white blood cell.
 
^*∗*^Details of criteria not described.

**Figure 1:**
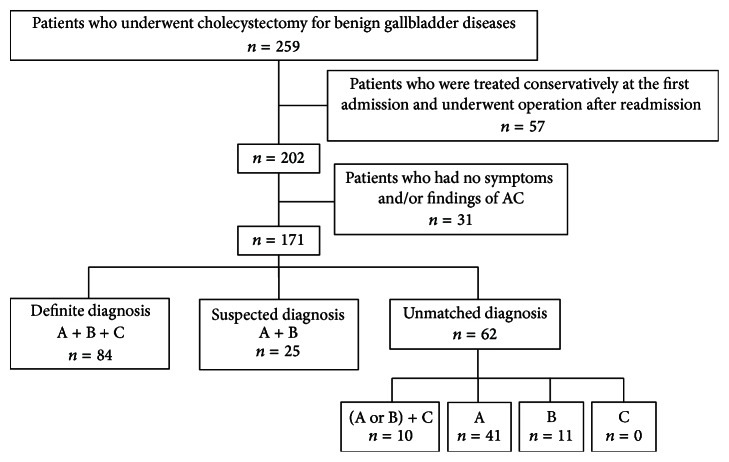
Flow diagram of the 259 patients who underwent cholecystectomy for benign gallbladder diseases according to the diagnostic criteria based on TG13. A, B, and C represent each item in the TG13 diagnostic criteria. AC: acute cholecystitis and TG13: Tokyo Guidelines 2013.

Figure 1 shows a flow diagram of the 259 patients who underwent cholecystectomy for benign gallbladder diseases. Of the subjects (171 patients), 84 patients were classified as having definite diagnosis (A + B + C); 25 suspected diagnosis (A + B); 62 unmatched diagnosis; 10 (A or B) + C; 41 A; and 11 B. No patients had only C.

### 2.2. Clinical Outcome Assessment

MC was calculated as the total amount of medical expenses during the hospital stay. To investigate the correlations between AC severity grading and MC, we used two different MC assessments: one was calculated by the fee-for-service (FFS) payment system and the other by the DPC system. The claim of medical expenses at our hospital was based on DPC/PDPS during the survey period.

### 2.3. Preoperative Factors Predicting Clinical Outcomes

To identify the preoperative factors predicting the clinical outcomes of patients with definite, suspected, or unmatched AC diagnoses, we accumulated preoperative clinical findings, including the severity grade based on TG13. These included (1) patient characteristics: age, sex, body mass index, history of diabetes mellitus, and PS; (2) preoperative diagnoses: gallbladder stones, CBD stones, acute cholangitis, and acute pancreatitis; (3) preoperative treatment: biliary drainage, hospital stay before surgery, and preoperative fasting period; (4) preoperative laboratory data: white blood cell count (WBC), hemoglobin, platelet cell count, C-reactive protein (CRP), serum albumin, blood urea nitrogen, serum creatinine, total bilirubin, aspartate aminotransferase, alanine aminotransferase, alkaline phosphatase, *γ*-glutamyl transpeptidase, serum amylase, and prothrombin time/international normalized ratio.

If patients had duplicate values in each item during the preoperative period, we selected the most unfavorable value for those patients. We determined the most useful preoperative factors to predict outcomes by using univariate and multivariate analyses.

### 2.4. Statistical Analysis

The data for continuous variables were expressed as mean values with standard deviations. The statistical significance of mean differences among the groups was determined by the Kruskal-Wallis test. In the evaluation of preoperative factors predicting clinical outcomes of AC (PHS and MC), simple regression analyses were first used to detect statistically significant associations between each preoperative factor and then a multiple linear regression analysis was used to identify the independent preoperative factors. Only factors that were statistically significant according to the univariate analysis were included in the multivariate analysis. The results were considered to be significant for values of *P* < 0.05.

## 3. Results

**Figure 2:**
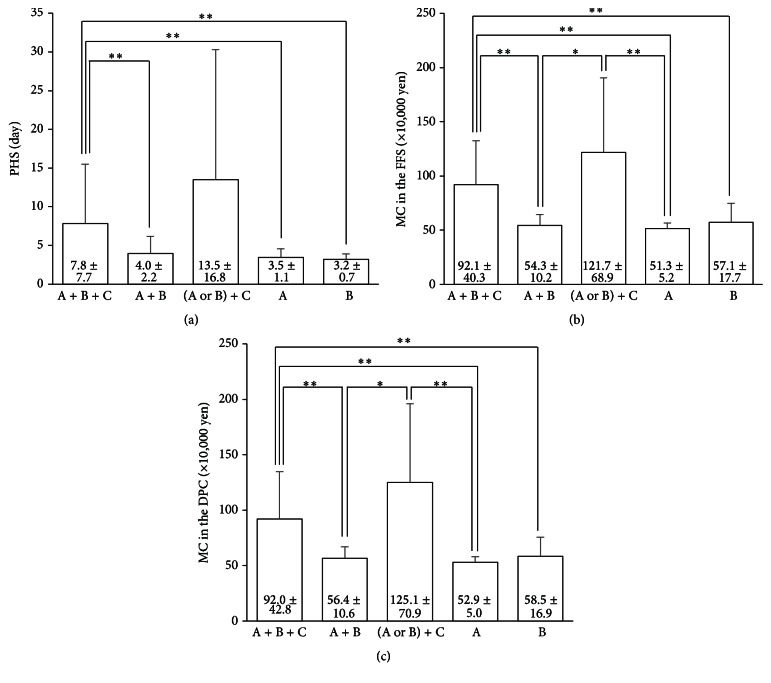
Comparison of PHS and MC in the FFS and DPC systems according to the five groups (*n* = 171). (a) The differences in the average PHS for each group. (b) The differences in the average MC in the FFS system for each group. (c) The differences in the average MC in the DPC system for each group. PHS: postoperative hospital stay, MC: medical costs, FFS: fee for service, and DPC: diagnosis procedure combination. ^*∗∗*^ *P* < 0.01 and ^*∗*^ *P* < 0.05.

The characteristics and medical backgrounds for 171 patients are shown in Table 1. Variables such as age, poor PS, WBC, CRP, acute cholangitis, hospital stay before surgery, and blood loss in the A + B + C and (A or B) + C groups were higher than those in the other groups. When we compared PHS and MC in the FFS system among the five groups of A + B + C (definite diagnosis), A + B (suspected diagnosis), (A or B) + C, A, and B, the A + B + C and (A or B) + C groups were equivalent, showing significantly higher values than the other groups (Figure 2). The backgrounds of 10 patients belonging to (A or B) + C were compared to those of 84 patients belonging to A + B + C. Regarding comorbidities in (A or B) + C, 5 patients (50%) had dementia (2 patients) or paralysis (3 patients). Among 84 patients in A + B + C, only 2 (2.4%) had dementia.

Regarding severity grade (Table 2), the 84 patients with definite diagnosis showed Grade I in 55 (65.5%), Grade II in 18 (21.4%), and Grade III in 11 (13.1%); all patients in A + B (*n* = 25), A (*n* = 41), and B (*n* = 11) belonged to Grade I. In contrast, among 10 patients with (A or B) + C, Grade I severity was noted in 7 (70%), Grade II in 2 (20%), and Grade III in 1 (10%). Postoperative complications with regard to severity grade for 171 patients are listed in Table 3. There was no postoperative mortality, and the incidence of postoperative complications significantly increased with severity grade: 4.3% in Grade I, 15.0% in Grade II, and 66.7% in Grade III. The incidence of preoperative drainage in Grade III was significantly higher than that in Grade I: 66.7% (8/12) versus 12.1% (15/124) (*P* < 0.001).

**Figure 3:**
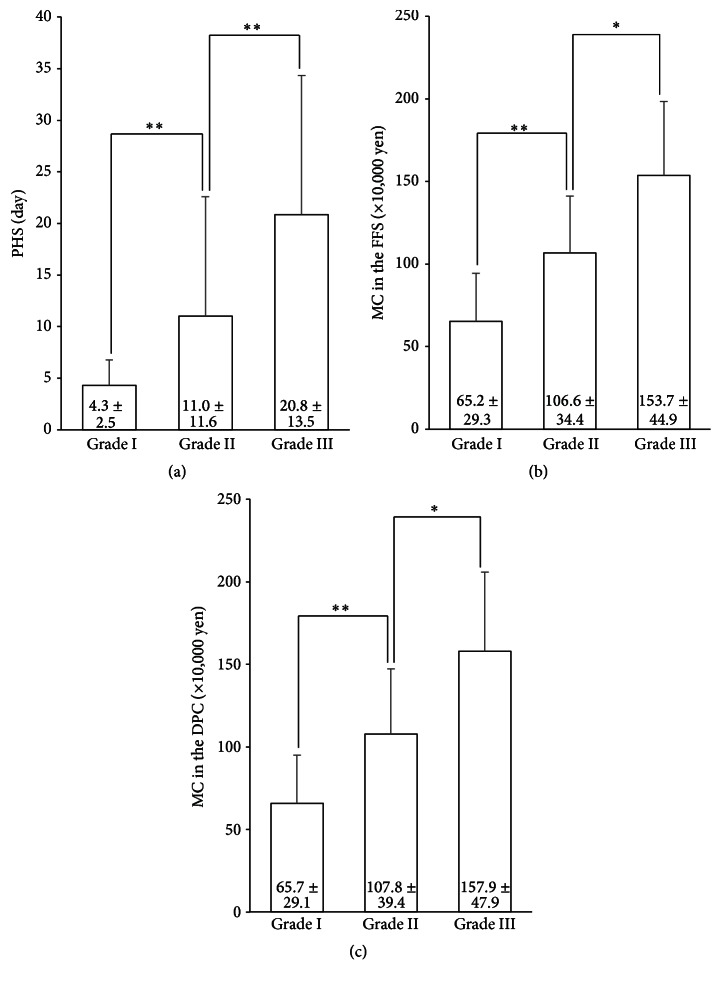
Comparison of PHS and MC according to severity grade (*n* = 171). (a) The differences in the average PHS for each grade. (b) The differences in the average MC in the FFS system for each grade. (c) The differences in the average MC in the DPC system for each grade. PHS: postoperative hospital stay, MC: medical costs, FFS: fee for service, and DPC: diagnosis procedure combination. ^*∗∗*^ *P* < 0.01 and ^*∗*^ *P* < 0.05.

**Figure 4:**
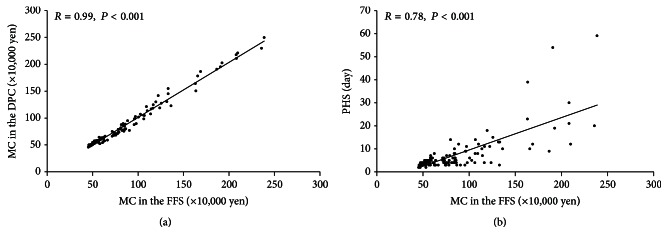
(a) Relationship between MC in the FFS system and MC in the DPC system (*n* = 171). (b) Relationship between PHS and MC in the FFS system (*n* = 171). PHS: postoperative hospital stay, MC: medical costs, FFS: fee for service, and DPC: diagnosis procedure combination.

When we compared PHS and MC (×10,000 yen) in the FFS and DPC systems according to severity grades based on TG13 (Figure 3), PHS and MC were significantly stratified for each grade. MC in the DPC system was strongly correlated with MC in the FFS system (*R* = 0.99, *P* < 0.001) (Figure 4(a)). Furthermore, we found that PHS was significantly correlated with MC in the FFS system (*R* = 0.78, *P* < 0.001), although some patients had markedly longer or shorter PHS compared to MC (Figure 4(b)). Therefore, we performed univariate and multivariate analyses of preoperative factors influencing PHS and MC.

Univariate analysis revealed the 21 preoperative factors significantly correlated with PHS as shown in Table 4. Interestingly, among three signs of A, B, and C for AC diagnosis, B (*P* = 0.004) and C (*P* < 0.001) were significantly associated with prolonged PHS. Multivariate analysis using these 21 factors identified the three factors of PS, WBC, and severity grade based on TG13 as significant independent preoperative factors influencing PHS (Table 5). Regarding MC, univariate analysis revealed the 24 preoperative factors significantly correlated with MC, and multivariate analysis using these 24 factors identified the six factors of age, PS, preoperative biliary drainage, hospital stay before surgery, albumin, and severity grade based on TG13 as significant independent preoperative factors influencing MC (Table 6).

## 4. Discussion

AC severity grading in TG13 focuses on the presence of organ dysfunction and is considered to predict severity accurately. When AC becomes more severe, it increases the risk of major complications and results in prolonged PHS [3]. However, no reports have evaluated the usefulness of the TG13 severity grading of AC in terms of PHS and MC. Our results showed that PHS and MC were significantly increased in the order of severity grades, although our study included patients with definite, suspected, or unmatched AC diagnoses. In order to reduce MC, DPC was introduced in 2003 in Japan [18]. Recent database analyses comparing the DPC and FFS systems for patients with acute myocardial infarction revealed that the DPC system significantly reduced total accumulated medical charges [32]. In the present study, however, a linear correlation (*R* = 0.99, *P* < 0.001) between MC in the DPC and FFS systems was observed, which in turn demonstrated that the DPC system in this cohort of patients did not reduce MC.

In contrast, the relationship between PHS and MC evaluated by the FFS system did not show a linear correlation, because some patients had markedly longer or shorter PHS compared to MC as shown in Figure 4(b). In order to clarify the significant preoperative factors influencing PHS, we performed univariate and multivariate analyses, which identified three significant independent factors: PS, WBC, and severity grade based on TG13. Cheng et al. assessed the impact of TG13 and the presence of comorbidities on clinical outcomes in 103 patients with AC by univariate and multivariate regression analyses [3]. According to multivariate analysis, patients with Grade III in TG13, higher Charlson's comorbidity scores (assessing the prognostic burden of comorbid disease), and postoperative complications had longer hospital stays. Furthermore, Murata et al. performed a large database analysis using Japanese DPC data in 2176 patients with AC in 2008, and multiple linear regression analyses revealed that early and laparoscopic cholecystectomy was significantly associated with a decrease in length of stay, whereas severity of comorbid conditions, age ≥ 80 years, intensive care unit use, longer pre- and postoperative antimicrobial therapy, and gallbladder drainage were significantly associated with an increase in length of hospital stay [11]. According to these previous studies, preoperative factors influencing length of hospital stay are severity grade based on TG13, severity of comorbid conditions, age, preoperative antimicrobial therapy, and gallbladder drainage. Severity of comorbid conditions including Charlson's comorbidity score, which includes the presence of hemiplegia and dementia, is associated with PS. No reports have evaluated the significance of PS in the length of hospital stay in patients with AC. According to an international prospective study of 460 elderly patients with cancer, PS was significantly associated with extended hospital stay [33]. Interestingly, in the present study, PS was not identified as a significant independent factor predicting PHS when we classified PS 0, 1, and 2 as “better” and PS 3 and 4 as “poor.” This indicated that the difference between the patients who were up and about more than 50% of waking hours (PS 3) and those who were confined to bed or a chair more than 50% of waking hours (PS 4) significantly influences PHS. WBC is a significant prognostic factor of AC [2, 34, 35]; therefore, an increased WBC is associated with prolonged PHS.

On the other hand, we revealed that significant independent preoperative factors influencing MC were age, PS, preoperative biliary drainage, hospital stay before surgery, albumin, and severity grade based on TG13. PS and severity grade were identified as significant independent factors influencing PHS and MC. Although we can understand that preoperative biliary drainage and hospital stay before surgery were associated with increased MC, it is difficult to explain why increased age and decreased albumin were significant independent factors, because there are no previous reports of MC analysis in AC. We speculate that elderly patients require more medical resources including drugs and that decreased albumin levels are associated with poor nutrition, which may increase MC.

With regard to AC diagnostic criteria, our study suggests that imaging findings (C) are essential for diagnosis, because the patients belonging to A + B + C and (A or B) + C were equivalent in PHS and MC, and univariate analysis revealed that B and C (*P* < 0.001) were significantly associated with prolonged PHS. Some patients who are elderly and/or have dementia or paralysis are unlikely to present with local signs of inflammation (A) or systemic signs of inflammation (B) [21] because of their decreased antistress capacity, especially the elderly [22, 23]. In the elderly with AC, the significance of ultrasound examination prior to cholecystectomy is emphasized because ultrasound score can accurately determine AC severity and may be used as a reference for surgical intervention timing and mode selection to guide clinical therapy [24]. Therefore, we recommend reassessment of the TG13 diagnostic criteria by taking the elderly and/or those with dementia into consideration.

## 5. Conclusions

PS and severity grade based on TG13 significantly influence prolonged PHS as well as increased MC in patients with definite, suspected, or unmatched AC diagnosis, and we have to pay attention to the elderly and those with dementia who may show AC findings on imaging studies but lack local or systemic signs of inflammation.

## Figures and Tables

**Table 1:** Characteristics and medical backgrounds for 171 patients who had definite, suspected, or unmatched AC diagnoses.

Variables	Total (*n* = 171)	A + B + C (*n* = 84)	A + B (*n* = 25)	(A or B) + C (*n* = 10)	A (*n* = 41)	B (*n* = 11)
Age (years)	64.2 ± 14.7	68.9 ± 13.5	58.1 ± 15.0	71.4 ± 17.9	57.2 ± 12.2	61.7 ± 10.0
Sex (male/female)	90/81	48/36	9/16	8/2	20/21	5/6
BMI (kg/m^2^)	23.7 ± 3.3	23.6 ± 3.3	25.0 ± 3.7	22.6 ± 3.2	23.7 ± 3.3	23.6 ± 3.1
DM (yes/no)	23/148	11/73	1/24	3/7	7/34	1/10
PS (0, 1/2/3/4)	87/36/20/20	28/19/17/20	22/2/0/1	2/2/1/5	27/10/2/2	8/3/0/0
CT scan (yes/no)	171/0	84/0	25/0	10/0	41/0	11/0
Gallbladder stones (yes/no)	171/0	84/0	24/1	9/1	41/0	11/0
CBD stones (yes/no)	22/149	16/68	2/23	3/7	0/41	0/11
WBC (×10^3^/*μ*L)	10.1 ± 5.2	13.6 ± 4.8	6.7 ± 1.9	11.0 ± 4.3	5.6 ± 1.1	7.2 ± 2.4
Alb (g/dL)	3.7 ± 0.8	3.3 ± 0.7	4.2 ± 0.3	3.0 ± 1.1	4.2 ± 0.4	3.9 ± 0.5
CRP (mg/dL)	6.6 ± 9.3	12.1 ± 10.2	0.9 ± 1.2	8.6 ± 7.9	0.1 ± 0	0.3 ± 0.3
Definitive diagnosis of AC (yes/no)	84/87	84/0	0/25	0/10	0/41	0/11
Acute cholangitis (yes/no)	29/142	24/60	2/23	3/7	0/41	0/11
Preoperative biliary drainage (yes/no)	28/143	11/73	5/20	2/11	6/35	4/4
Hospital stay before surgery (day)	4.6 ± 6.2	6.9 ± 7.0	1.3 ± 1.0	9.2 ± 7.8	1.2 ± 0.8	2.7 ± 5.2
Surgical procedure (laparoscopic/conversion to open/open)	147/20/4	66/15/3	24/1/0	7/2/1	39/2/0	11/0/0
Length of operation (min)	108 ± 34	121 ± 33	98 ± 25	125 ± 49	89 ± 22	89 ± 23
Blood loss (mL)	152 ± 291	243 ± 310	14 ± 40	428 ± 585	18 ± 38	31 ± 39
Histopathology of gallbladder (cases)						
AC	52	47	0	4	1	0
Chronic cholecystitis	117	35	25	6	40	11
Other	2	2	0	0	0	0

AC: acute cholecystitis, BMI: body mass index, DM: diabetes mellitus, PS: performance status (Eastern Cooperative Oncology), CT: computed tomography, CBD: common bile duct, WBC: white blood cell, Alb: albumin, and CRP: C-reactive protein.

**Table 2:** Distribution according to severity grades based on TG13 in the 171 patients who had definite, suspected, or unmatched AC diagnoses.

Combination of criteria	Grade I (*n* = 139)	Grade II (*n* = 20)	Grade III (*n* = 12)
Definite diagnosis			
A + B + C (*n* = 84)	55 (65.5%)	18 (21.4%)	11 (13.1%)
Suspected diagnosis			
A + B (*n* = 25)^*∗*^	25 (100%)	0	0
Unmatched diagnosis			
(A or B) + C (*n* = 10)^*∗*^	7 (70.0%)	2 (20.0%)	1 (10.0%)
A (*n* = 41)^*∗*^	41 (100%)	0	0
B (*n* = 11)^*∗*^	11 (100%)	0	0

^*∗*^These patients were also classified according to severity grades based on TG13 as a matter of convenience.

TG13: Tokyo Guidelines 2013 and AC: acute cholecystitis.

**Table 3:** Postoperative complications according to severity grade in the 171 patients who had definite, suspected, or unmatched AC diagnoses.

Complications	Grade I (*n* = 139)	Grade II (*n* = 20)	Grade III (*n* = 12)
Urinary tract infection	0	0	1
Acute cholangitis	0	0	1
Decreased activities of daily living	0	0	3
Surgical site infection	1	1	0
Loss of appetite	1	0	2
Aspiration pneumonia	0	1	0
Deep vein thrombosis	1	0	0
Delirium	0	1	0
Other	2	0	0

Total (*n* = 17)	6 (4.3%)	3 (15.0%)	8 (66.7%)

AC: acute cholecystitis.

**Table 4:** Univariate analyses of the factors influencing PHS in the 171 patients who had definite, suspected, or unmatched AC diagnoses.

Variables	Regression coefficient	*P* value
Age	0.205	<0.001^*∗∗*^
Sex	2.168	0.055
BMI	−0.374	0.025^*∗*^
DM	1.632	0.325
PS	9.920	<0.001^*∗∗*^
Gallbladder stone	−12.911	0.013^*∗*^
CBD stone	4.472	0.008^*∗∗*^
Acute cholangitis	3.199	0.033^*∗*^
Acute pancreatitis	−2.350	0.381
Preoperative biliary drainage	5.949	<0.001^*∗∗*^
Hospital stay before surgery	0.414	<0.001^*∗∗*^
Preoperative fasting period	0.679	<0.001^*∗∗*^
WBC	0.556	<0.001^*∗∗*^
Hb	−1.503	<0.001^*∗∗*^
Plt	−0.035	<0.001^*∗∗*^
CRP	0.315	<0.001^*∗∗*^
Alb	−4.832	<0.001^*∗∗*^
BUN	0.244	<0.001^*∗∗*^
Cr	1.222	0.110
T-Bil	1.667	<0.001^*∗∗*^
AST	0.001	0.544
ALT	0.002	0.523
ALP	0.004	0.018^*∗*^
*γ*-GTP	−0.0001	0.947
AMY	0.0000	0.973
PT-INR	19553	<0.001^*∗∗*^
Severity grade based on TG13	7.859	<0.001^*∗∗*^
A for the diagnosis of AC based on TG13	−2.632	0.143
B for the diagnosis of AC based on TG13	3.707	0.004^*∗∗*^
C for the diagnosis of AC based on TG13	4.830	<0.001^*∗∗*^

PHS: postoperative hospital stay, AC: acute cholecystitis, BMI: body mass index, DM: diabetes mellitus, PS: performance status, CBD: common bile duct, WBC: white blood cell, Hb: hemoglobin, Plt: platelet cell, CRP: C-reactive protein, Alb: albumin, BUN: blood urea nitrogen, Cr: creatinine, T-Bil: total bilirubin, AST: aspartate aminotransferase, ALT: alanine aminotransferase, ALP: alkaline phosphatase, *γ*-GTP: *γ*-glutamyl transpeptidase, AMY: serum amylase, and PT-INR: prothrombin time-international normalized ratio. ^*∗∗*^ *P* < 0.01 and ^*∗*^ *P* < 0.05.

**Table 5:** Multivariate analyses of the factors influencing PHS in the 171 patients who had definite, suspected, or unmatched AC diagnoses.

Variables	Regression coefficient	*P* value
Age	0.0579	0.1106
BMI	0.0638	0.6531
*PS*	4.4929	0.0022^*∗∗*^
Gallbladder stone	−2.7409	0.5475
CBD stone	2.1809	0.3002
Acute cholangitis	−1.9900	0.2351
Preoperative biliary drainage	−0.7287	0.6988
Hospital stay before surgery	−0.0768	0.4268
Preoperative fasting period	0.1777	0.2807
*WBC*	−0.4312	0.0107^*∗*^
Hb	0.3093	0.4013
Plt	0.0007	0.9304
CRP	−0.0379	0.6538
Alb	−1.8506	0.1447
BUN	0.0855	0.0567
T-Bil	0.4488	0.3045
ALP	−0.0029	0.1186
PT-INR	1.3707	0.7111
*Severity grade based on TG13*	6.6843	<0.001^*∗∗*^
B for the diagnosis of AC based on TG13	0.6840	0.5722
C for the diagnosis of AC based on TG13	1.3101	0.3456

PHS: postoperative hospital stay, AC: acute cholecystitis, BMI: body mass index, PS: performance status, CBD: common bile duct, WBC: white blood cell, Hb: hemoglobin, Plt: platelet cell, CRP: C-reactive protein, Alb: albumin, BUN: blood urea nitrogen, T-Bil: total bilirubin, ALP: alkaline phosphatase, PT-INR: prothrombin time-international normalized ratio, and TG13: Tokyo Guidelines 2013. ^*∗∗*^ *P* < 0.01 and ^*∗*^ *P* < 0.05.

**Table 6:** Multivariate analyses of the factors influencing MC in the FFS system in the 171 patients who had definite, suspected, or unmatched AC diagnoses.

Variables	Regression coefficient	*P* value
*Age*	0.3518	0.0050^*∗∗*^
BMI	0.1676	0.7263
*PS*	19.7213	<0.001^*∗∗*^
CBD stone	2.2151	0.7495
Acute cholangitis	−2.7459	0.6357
*Preoperative biliary drainage*	13.5827	0.0320^*∗*^
*Hospital stay before surgery*	2.6646	<0.001^*∗∗*^
Preoperative fasting period	0.9821	0.0586
WBC	−0.5084	0.3568
Hb	1.0317	0.4094
Plt	0.0169	0.5015
CRP	−0.0842	0.7555
*Alb*	−10.1145	0.0197^*∗*^
BUN	0.0705	0.7116
Cr	1.1566	0.6605
T-Bil	0.4788	0.7585
AST	−0.0126	0.3581
ALT	0.0181	0.3973
ALP	−0.0122	0.0719
*γ*-GTP	0.0041	0.5503
PT-INR	9.1888	0.4562
*Severity grade based on TG13*	14.4135	<0.001^*∗∗*^
B for the diagnosis of AC based on TG13	−0.8822	0.8311
C for the diagnosis of AC based on TG13	2.1487	0.6437

MC: medical costs, FFS: fee for service, AC: acute cholecystitis, BMI: body mass index, PS: performance status, CBD: common bile duct, WBC: white blood cell, Hb: hemoglobin, Plt: platelet cell, CRP: C-reactive protein, Alb: albumin, BUN: blood urea nitrogen, Cr: creatinine, T-Bil: total bilirubin, AST: aspartate aminotransferase, ALT: alanine aminotransferase, ALP: alkaline phosphatase, *γ*-GTP: *γ*-glutamyl transpeptidase, PT-INR: prothrombin time-international normalized ratio, and TG13: Tokyo Guidelines 2013. ^*∗∗*^ *P* < 0.01 and ^*∗*^ *P* < 0.05.
